# Joint fibrosis research from a multi-joint perspective: a bibliometric analysis, 2000–2025

**DOI:** 10.3389/fsurg.2026.1885682

**Published:** 2026-09-16

**Authors:** Lexin Chen, Guoyan Wang, Ge Lei, Yuzhi Jiang, Rui Huang, Dingqun Bai, Weiwei Yi

**Affiliations:** Department of Rehabilitation Medicine, Key Laboratory of Physical Medicine and Precision Rehabilitation of Chongqing Municipal Health Commission, The First Affiliated Hospital of Chongqing Medical University, Chongqing, China

**Keywords:** author keywords, bibliometrics, collaboration networks, joint fibrosis, knee

## Abstract

**Background:**

Joint fibrosis is a pathological process characterized by abnormal tissue repair, excessive extracellular matrix deposition, and fibrous adhesions. It can affect multiple joints and cause pain, restricted motion, and functional impairment. Existing research has largely focused on specific joints or surgical procedures, and the overall research landscape and literature distribution across joints have not been systematically characterized.

**Methods:**

Scopus and the Web of Science Core Collection were searched using a strategy that combined a fibrosis-specific concept module with a joint anatomy module. After restriction, merging, and deduplication, 1,600 English-language articles and reviews were included. Bibliometric analyses were performed in R using bibliometrix, Biblioshiny, and ggplot2.

**Results:**

Publication output on joint fibrosis increased overall from 2000 to 2025, with an annual growth rate of 9.81%; annual publications rose from 19 in 2000 to 197 in 2025. Author keywords centered on arthrofibrosis and were closely associated with total knee arthroplasty, anterior cruciate ligament reconstruction, joint stiffness, postoperative complications, and functional recovery. Anatomical classification identified 1,143 knee-related publications, substantially more than for any other joint. Collaboration networks comprised several relatively concentrated author and institutional groups, while the United States occupied a central position in publication output, international collaboration, and the country collaboration network.

**Conclusion:**

Within the available bibliographic data, joint fibrosis research was highly concentrated on the knee and focused primarily on joint arthroplasty, ligament reconstruction, and postoperative functional management. Future research should adopt standardized definitions and outcome measures, expand investigation to underrepresented joints, and integrate mechanistic and prospective clinical studies to improve the diagnosis, prevention, and management of joint fibrosis.

## 1 Introduction

Joint fibrosis is an abnormal tissue-repair process characterized by persistent activation of fibroblasts and myofibroblasts, excessive extracellular matrix deposition, and fibrous adhesions within the joint or joint capsule. It may develop after trauma, surgery, infection, recurrent inflammation, or prolonged immobilization. Persistent inflammatory and profibrotic signaling disrupts the normal termination of tissue repair, promotes collagen deposition, scar formation, and soft-tissue contracture, and ultimately causes pain, reduced range of motion, and functional impairment (1–5).

Joint fibrosis is a pathological concept, whereas stiffness and contracture primarily describe clinical manifestations; these terms are not interchangeable. In addition to fibrous adhesions and capsular fibrosis, restricted joint motion may arise from pain, heterotopic ossification, osseous impingement, component malposition, infection, or neuromuscular disorders. Previous studies have also used inconsistent diagnostic thresholds, severity classifications, and criteria for excluding alternative causes, further complicating comparisons across studies (6). An expert consensus on the knee emphasized that postoperative joint fibrosis should be diagnosed only after excluding osseous or prosthetic mechanical obstruction, infection, pain, and other identifiable causes (7). Therefore, using stiffness or contracture alone as a search term or diagnostic criterion may unduly broaden the boundaries of joint fibrosis research.

Joint fibrosis can involve the knee, shoulder, elbow, hip, hand, foot, temporomandibular joint, and other sites, but these joints differ in injury context, anatomy, mechanical environment, and outcome assessment. Knee research commonly concerns total knee arthroplasty, anterior cruciate ligament reconstruction, and complex trauma; clinical management includes rehabilitation, manipulation under anesthesia, arthroscopic lysis of adhesions, and revision surgery (8–11). Capsular fibrosis of the shoulder may involve fibroblast activation and dysregulated inflammatory mediators, but frozen shoulder and postoperative shoulder stiffness are not necessarily equivalent to pathologically confirmed joint fibrosis (12–14). Post-traumatic restriction of elbow motion may simultaneously involve capsular contracture, intra-articular adhesions, heterotopic ossification, and osseous impingement (15, 16). Fibrous adhesions or capsular disorders of the hip, foot, and temporomandibular joint have also been reported, although these studies are generally dispersed across specialties (17–21).

Bibliometrics uses structured bibliographic data to characterize research output, source journals, authors, institutions, countries, and citation patterns. Science mapping can reveal the thematic and social structures of a field through keyword co-occurrence, conceptual structures, and collaboration networks (22–25). For joint fibrosis, which spans orthopedics, sports medicine, rehabilitation medicine, and basic fibrosis research, these approaches can identify the relative visibility of themes and anatomical sites in the available literature. However, the validity of bibliometric findings depends heavily on corpus boundaries. Treating general joint stiffness, postoperative stiffness, joint contracture, frozen shoulder, or adhesive capsulitis as independent search entry points could retrieve many records without explicit evidence of fibrosis and systematically bias the corpus toward particular joints or clinical phenotypes.

Accordingly, this study used a retrieval framework combining a fibrosis-specific concept module with a joint anatomy module to perform a bibliometric and science mapping analysis of joint fibrosis literature indexed in Scopus and the Web of Science Core Collection from 2000 to 2025. We aimed to describe changes in annual output and citations; identify leading journals, authors, institutions, and countries; analyze author keywords, thematic structures, and collaboration networks; and compare the relative visibility of different joints in the bibliographic data.

## 2 Methods

### 2.1 Study design

This bibliometric and science mapping study was designed to characterize publication output, bibliographic impact, knowledge structures, anatomical distributions, and collaboration patterns in joint fibrosis research. It used publicly available bibliographic metadata and did not synthesize clinical outcomes, evaluate intervention effects, or grade evidence quality. Therefore, conventional methods for clinical systematic reviews, including the PICOS framework, risk-of-bias assessment, and effect-size synthesis, were not applied.

### 2.2 Literature search and data integration

Scopus and the Web of Science Core Collection (WoS) were selected as data sources. Both databases provide broad multidisciplinary coverage and structured bibliographic information, including authors, affiliations, author keywords, cited references, and citation counts, making them suitable for bibliometric analysis and science mapping. Searching both databases helped reduce potential bias arising from the coverage of a single database.

The search strategy comprised a fibrosis-specific concept module and a joint anatomy module (Table 1). The fibrosis module included terms explicitly denoting fibrosis or fibrous adhesions, such as arthrofibrosis, joint fibrosis, capsular fibrosis, synovial fibrosis, and fibrous adhesion. The anatomy module was based on the MeSH hierarchy for Joints and covered the knee, shoulder, elbow, hip, hand, foot, temporomandibular, sacroiliac, and other joints, supplemented by relevant synonyms, spelling variants, and common abbreviations (Supplementary Table S1). Terms that do not independently establish a fibrotic pathological process—such as joint stiffness, postoperative stiffness, joint contracture, frozen shoulder, and adhesive capsulitis—were not used as standalone inclusion criteria. Searches were conducted in TITLE-ABS-KEY in Scopus and the TS topic field in WoS.

**Table 1:** Search framework for joint fibrosis.

Component	Search terms or description
Fibrosis concept block (A)	(arthrofibros* OR “joint fibrosis” OR “articular fibrosis” OR “intra-articular fibrosis” OR “intraarticular fibrosis” OR “periarticular fibrosis” OR “peri-articular fibrosis” OR “joint capsule fibrosis” OR “capsular fibrosis” OR “fibrosis of the joint capsule” OR “synovial fibrosis” OR “fibrosis of the synovium” OR “fibrotic joint capsule” OR “intra-articular fibrous adhesion*” OR “intraarticular fibrous adhesion*” OR “fibrous intra-articular adhesion*” OR “intra-articular fibrous scar*”)
Joint anatomy block (B)	(joint* OR shoulder OR glenohumeral OR “acromioclavicular joint*” OR acromioclavicular OR elbow OR “elbow joint*” OR wrist OR radiocarpal OR “hand joint*” OR “finger joint*” OR metacarpophalangeal OR interphalangeal OR hip OR “hip joint*” OR acetabulofemoral OR coxofemoral OR knee OR “knee joint*” OR tibiofemoral OR patellofemoral OR ankle OR talocrural OR tibiotalar OR subtalar OR “foot joint*” OR “toe joint*” OR metatarsophalangeal OR temporomandibular OR “temporomandibular joint*” OR TMJ OR “atlanto-axial joint*” OR “atlantoaxial joint*” OR atlantoaxial OR “atlanto-occipital joint*” OR “atlantooccipital joint*” OR atlantooccipital OR “pubic symphysis” OR “symphysis pubis” OR “sacroiliac joint*” OR sacroiliac OR “sternoclavicular joint*” OR sternoclavicular OR “sternocostal joint*” OR sternocostal OR “zygapophyseal joint*” OR “facet joint*”)
Web of Science Core Collection	TS = (A AND B)
Scopus	TITLE-ABS-KEY(A AND B)

Records were converted to a unified bibliographic data format using convert2df() in the R package bibliometrix, and within-database duplicates were removed using database-specific unique record identifiers. The Scopus and WoS datasets were then merged using mergeDbSources() with remove.duplicated = TRUE to remove cross-database duplicates.

The literature search was completed on August 14, 2026. The initial Scopus search retrieved 1,988 records; restricting the publication years to 2000–2025 yielded 1,704 records. Further restriction to English-language articles and reviews at the final publication stage yielded 1,382 records. The initial WoS search retrieved 1,314 records, of which 1,082 remained after restrictions on publication year, document type, and language. After the two database exports were merged and deduplicated in R, the final analytical dataset contained 1,600 unique publications (Figure 1). This process comprised database searching, restriction, and data integration and was not equivalent to independent title, abstract, or full-text eligibility screening in a conventional systematic review.

**Figure 1:**
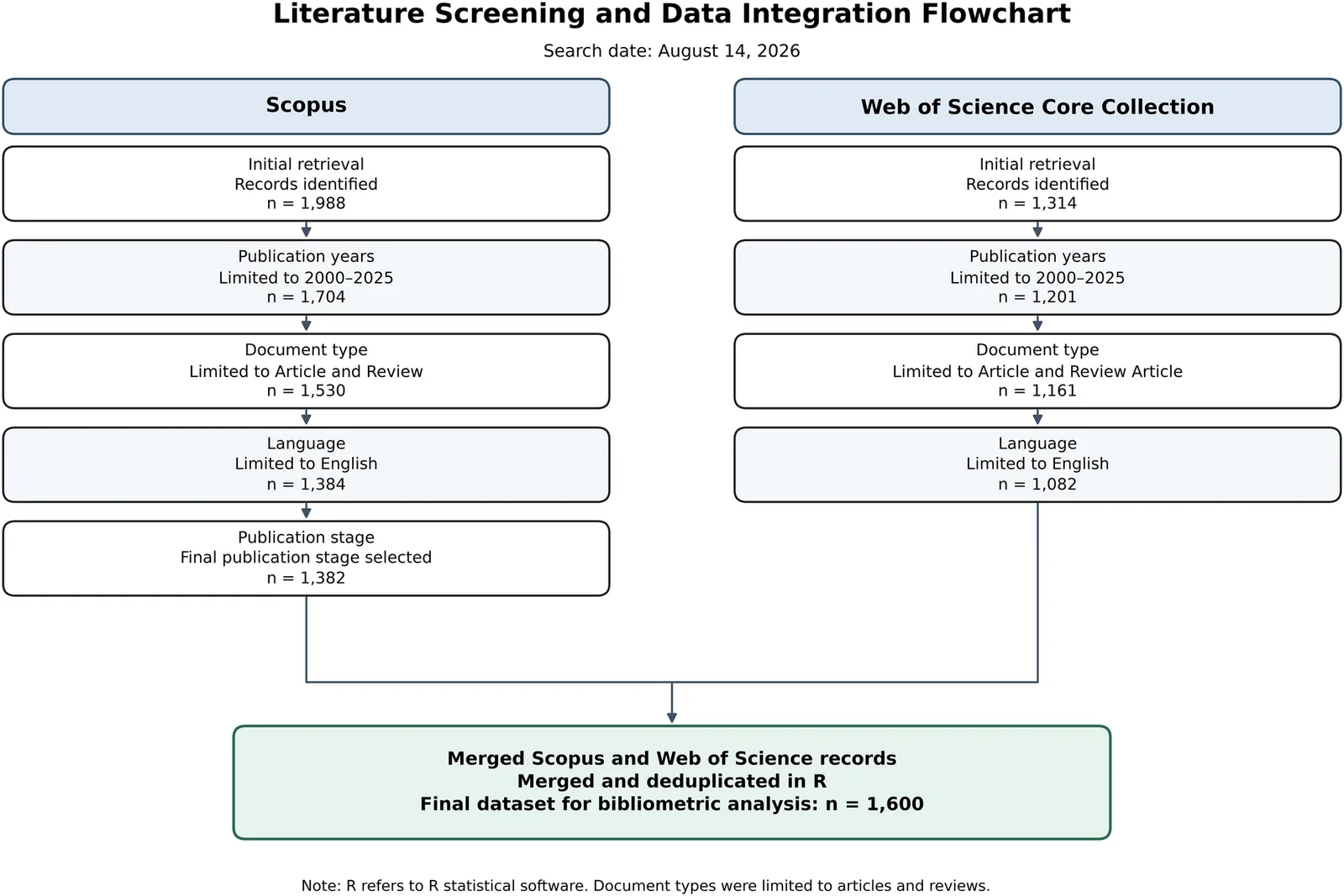
Literature search and data integration flowchart. The flowchart shows the searching, restriction, merging, and deduplication of records from Scopus and the Web of Science Core Collection, resulting in the inclusion of 1,600 publications.

### 2.3 Author identity resolution and institutional name processing

To reduce erroneous merging of authors with abbreviated names, DOI and OpenAlex data were used to supplement author identity resolution. Of the 1,600 publications, 1,527 had a DOI; 1,519 were successfully matched in OpenAlex and eight were unmatched. Another 73 publications lacked a DOI, leaving 81 publications without an OpenAlex match. For matched publications, authors were identified primarily by OpenAlex Author ID, with ORCID used as a secondary reference. Institutions were counted according to the signing entities recorded in the databases. Harvard University, Harvard Medical School, Harvard University Medical Affiliates, and related hospitals were not simply merged because they appeared as separate affiliations in the bibliographic records.

### 2.4 Bibliometric analysis

Bibliometric analyses were conducted primarily using the R package bibliometrix and its visualization interface, Biblioshiny; selected results were redrawn with ggplot2 (26, 27). Analyses covered annual publications and citations, source journals, authors, institutions, countries, author keywords, and collaboration. Journal-, country-, and citation-based indicators were calculated only from the 1,600 publications included in this study and do not represent overall evaluations of the corresponding journals or countries. Keyword frequency, temporal dynamics, co-occurrence networks, conceptual structure, and trend-topic analyses used only author keywords, excluding WoS Keywords Plus and index keywords automatically generated by Scopus.

### 2.5 Anatomical classification of joints

The joint anatomy dictionary was based on the MeSH hierarchy for Joints and supplemented with free-text names, anatomical synonyms, singular and plural forms, spelling variants, and common abbreviations found in the corpus (Supplementary Table S1). Each publication was classified by matching terms in its title, abstract, and author keywords. Multiple matches within the same joint category were counted once per publication, whereas publications involving multiple joints could be assigned to multiple categories. Consequently, categories were not mutually exclusive and their counts could exceed the corpus total. The wrist and interphalangeal joints were grouped as hand joints, while the ankle and metatarsophalangeal joints were grouped as foot joints; classification granularity was not identical across anatomical categories.

## 3 Results

### 3.1 Dataset characteristics and annual trends

The final corpus covered 2000–2025 and comprised 1,600 publications from 418 sources. The annual growth rate was 9.81%, the mean document age was 8.35 years, and the mean number of citations per publication was 24.19. The dataset contained 2,592 author keywords and 5,967 authors; the mean number of coauthors per publication was 5.77, and international coauthorship accounted for 14.81% of publications (Table 2). These findings indicate that multi-author collaboration was common, whereas international collaboration remained relatively limited.

**Table 2:** Overview of the final dataset used for bibliometric analysis.

Description	Results
Timespan	2000:2025
Sources (journals, books, etc.)	418
Documents	1,600
Annual growth rate %	9.81
Document average age	8.35
Average citations per doc	24.19
Keywords plus (ID)	5,224
Author's keywords (DE)	2,592
Authors	5,967
Authors of single-authored docs	43
Single-authored docs	53
Co-authors per doc	5.77
International co-authorships %	14.81

Annual publication output increased overall and accelerated after 2017, rising from 19 publications in 2000 to 197 in 2025 (Figure 2A; Table 3). The mean annual citation rate varied by publication year and was highest in 2002 at 4.22 citations; rates were relatively lower for recent publications, possibly because less time had elapsed for citations to accumulate (Figure 2B; Table 3). Annual publication output and citation performance did not increase in parallel, indicating that greater research activity was not consistently accompanied by higher citation impact. The rise in publication counts should therefore be interpreted as an increase in the absolute volume of research on joint fibrosis, potentially reflecting broader growth in scientific publishing, expanded database coverage, or increased research attention, rather than evidence that the field occupied a larger share of the scientific literature or that the clinical burden of joint fibrosis had increased.

**Figure 2:**
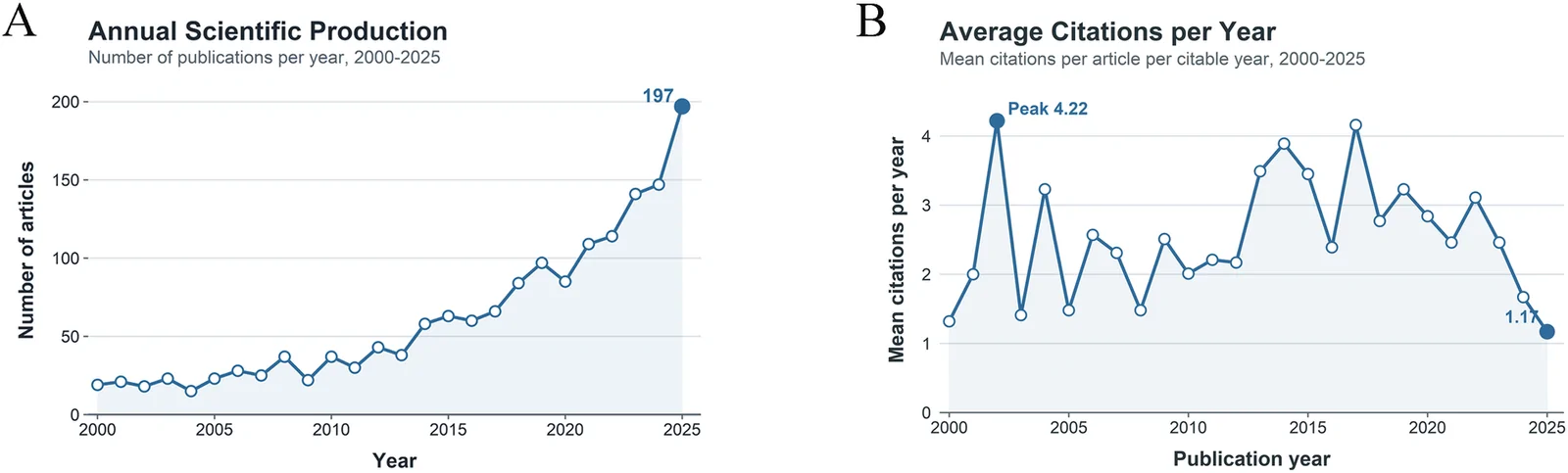
Annual publication and citation trends in joint fibrosis research, 2000–2025. **(A)** Annual publication output. **(B)** Mean annual citations per publication by publication year.

**Table 3:** Annual scientific production and average citations per year.

Year	No of articles	Mean TC per art	Mean TC per year	Citable years
2000	19	35.58	1.32	27
2001	21	51.95	2	26
2002	18	105.5	4.22	25
2003	23	33.74	1.41	24
2004	15	74.4	3.23	23
2005	23	32.61	1.48	22
2006	28	53.93	2.57	21
2007	25	46.12	2.31	20
2008	37	28.11	1.48	19
2009	22	45.23	2.51	18
2010	37	34.14	2.01	17
2011	30	35.4	2.21	16
2012	43	32.6	2.17	15
2013	38	48.92	3.49	14
2014	58	50.52	3.89	13
2015	63	41.35	3.45	12
2016	60	26.28	2.39	11
2017	66	41.56	4.16	10
2018	84	24.92	2.77	9
2019	97	25.82	3.23	8
2020	85	19.87	2.84	7
2021	109	14.77	2.46	6
2022	114	15.54	3.11	5
2023	141	9.84	2.46	4
2024	147	5.01	1.67	3
2025	197	2.34	1.17	2

### 3.2 Core journals and bibliographic impact

The literature was published mainly in journals specializing in joint arthroplasty, arthroscopy, sports medicine, and orthopedics. The Journal of Arthroplasty published the most articles (*n* = 104), followed by the Orthopaedic Journal of Sports Medicine (*n* = 64); Arthroscopy—The Journal of Arthroscopic and Related Surgery and Knee Surgery, Sports Traumatology, Arthroscopy each published 52 articles (Figure 3A). Cumulative output increased across the leading journals, with the most pronounced growth in the Journal of Arthroplasty (Figure 3B). Within the publications included in this study, the Journal of Arthroplasty ranked first for the h-index (34), g-index (62), m-index (1.31), and total citations (4,090) (Table 4), indicating high bibliographic impact for its joint fibrosis literature within this dataset.

**Figure 3:**
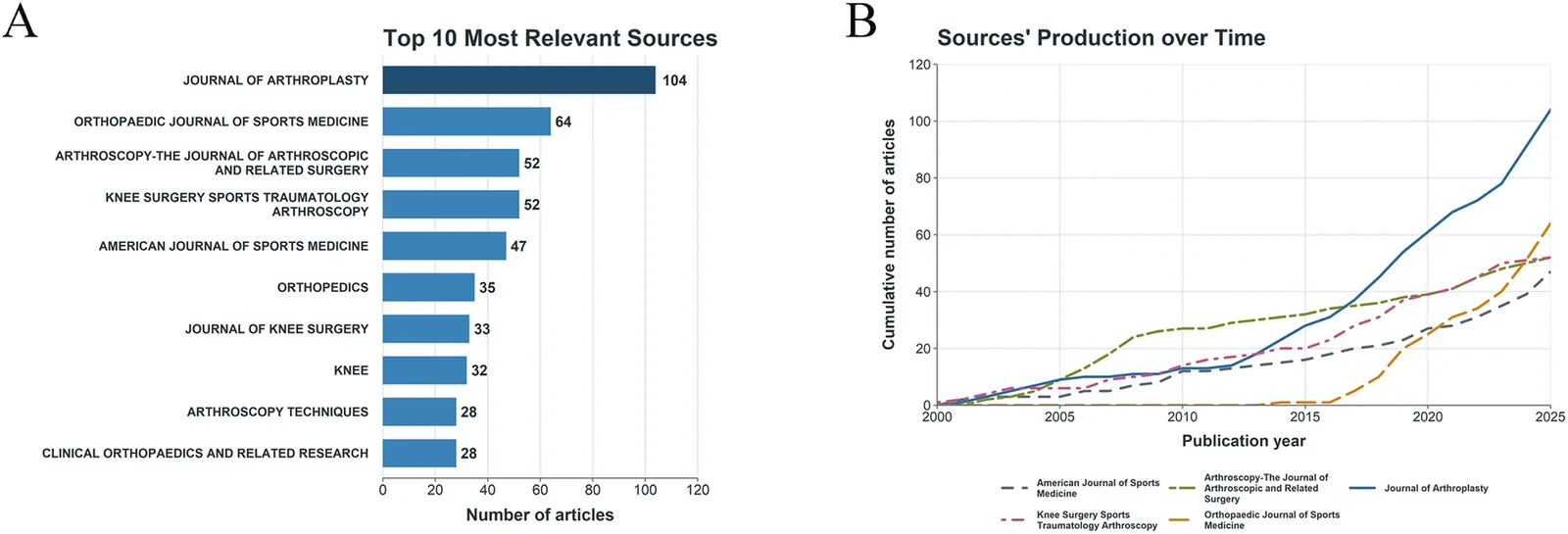
Leading source journals in joint fibrosis research. **(A)** The 10 journals with the highest publication output. **(B)** Cumulative publication trends of the top five journals.

**Table 4:** Top 5 journals by h-index, g-index, m-index, and total citations.

Source	h_index	g_index	m_index	Total citations
Journal of arthroplasty	34	62	1.31	4,090
Arthroscopy-the journal of arthroscopic and related surgery	27	44	1.08	2,016
American journal of sports medicine	26	44	1	1,988
Knee surgery sports traumatology arthroscopy	25	42	0.93	1,881
Clinical orthopaedics and related research	21	28	0.78	2,534

### 3.3 Distribution of authors, institutions, and countries

After author identity disambiguation, Matthew P. Abdel was the most prolific author with 47 publications, followed by Joaquín Sánchez-Sotelo (*n* = 33) and Mark E. Morrey (*n* = 32) (Figure 4A). Mayo Clinic ranked first among institutions with 155 publications, followed by Harvard University (*n* = 85), Harvard University Medical Affiliates (*n* = 67), and Harvard Medical School (*n* = 55) (Figure 4B).

**Figure 4:**
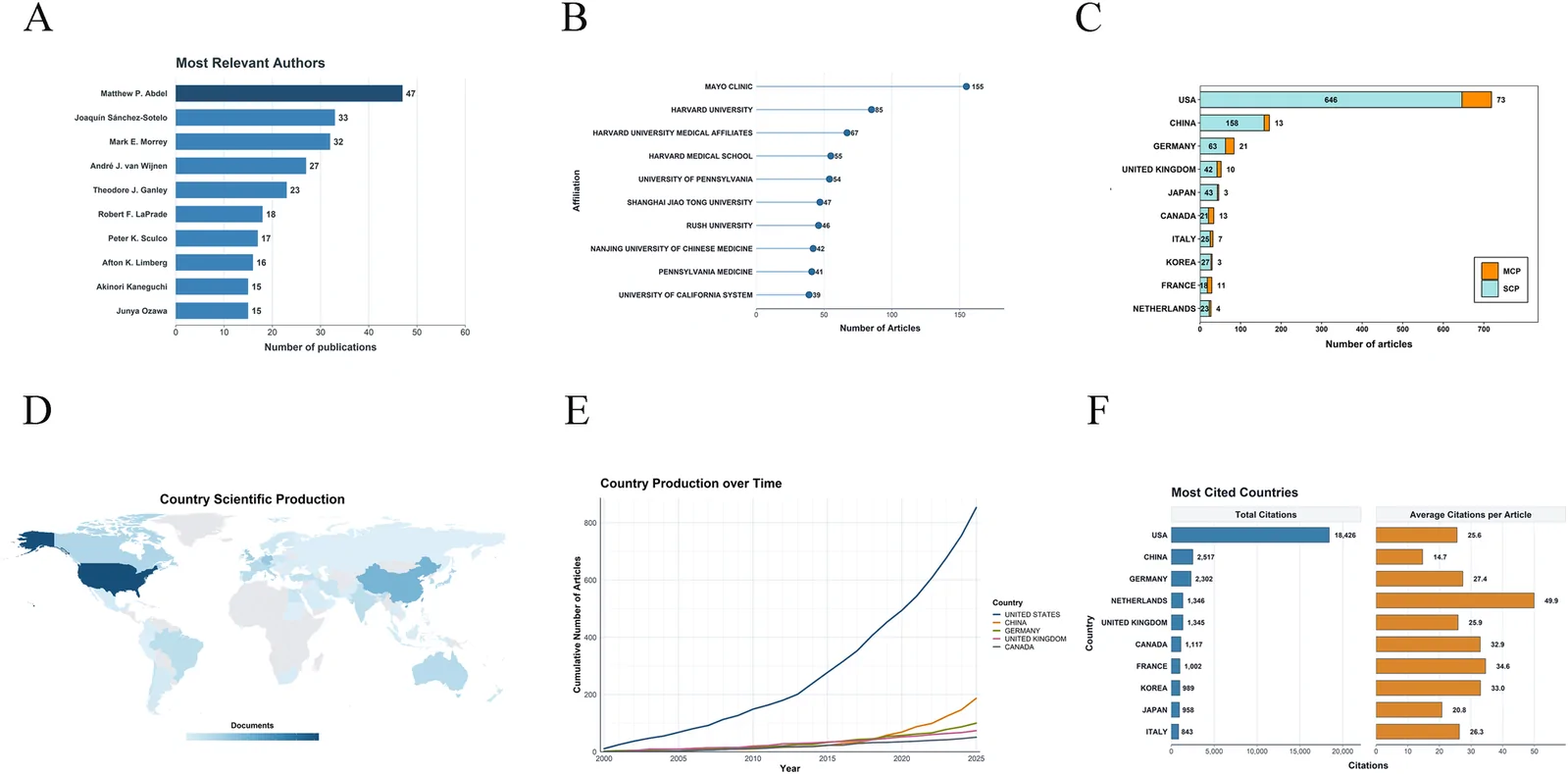
Distribution of authors, institutions, and countries. **(A)** The 10 most productive authors. **(B)** The 10 most productive institutions. **(C)** Single-country and multiple-country publications by corresponding-author country. **(D)** Global distribution of Country Scientific Production. **(E)** Cumulative trends in Country Scientific Production for the leading countries. **(F)** Total and mean citations for the leading cited countries. SCP, single-country publication; MCP, multiple-country publication.

Based on corresponding-author country, the United States contributed 719 publications, comprising 646 single-country publications (SCPs) and 73 multiple-country publications (MCPs). China ranked second with 171 publications, including 158 SCPs and 13 MCPs, followed by Germany with 84 publications (63 SCPs and 21 MCPs) (Figure 4C). When all author countries were considered using whole counting, the United States showed the highest scientific output, followed by China, Germany, the United Kingdom, and Canada (Figures 4D, E). The United States maintained a substantial lead throughout the study period, while China exhibited particularly rapid growth in recent years (Figure 4E).

The United States also received the highest total number of citations (*n* = 18,426), followed by China (*n* = 2,517) and Germany (*n* = 2,302). Among the countries displayed, the Netherlands had the highest average number of citations per article (49.9), followed by France (34.6), South Korea (33.0), and Canada (32.9) (Figure 4F).

### 3.4 Author keywords and anatomical distribution of joints

Arthrofibrosis was the most frequent author keyword (312 occurrences), followed by total knee arthroplasty (149), knee (143), and anterior cruciate ligament (85) (Figure 5A). The cumulative frequency of leading author keywords increased over time, most notably for arthrofibrosis, total knee arthroplasty, and knee (Figure 5D). The author-keyword structure linked joint fibrosis as the central concept to clinical topics involving knee arthroplasty, ligament injury and reconstruction, postoperative complications, joint stiffness, arthroscopic treatment, and recovery of range of motion.

**Figure 5:**
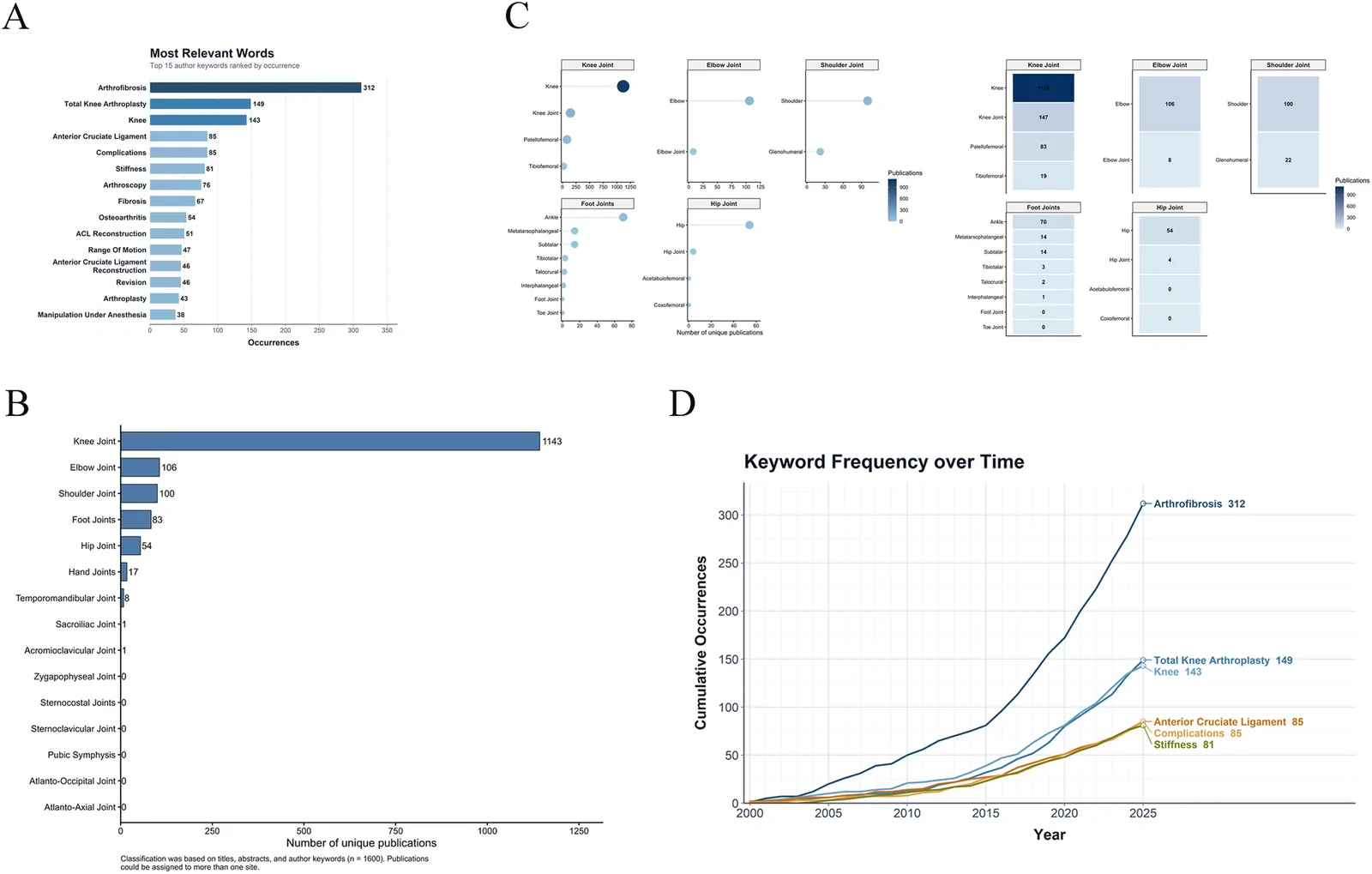
Author keywords and anatomical distribution of joints. **(A)** The 15 most frequent author keywords. **(B)** Number of unique publications assigned to each joint category. **(C)** Publication-level distribution of specific anatomical terms within the major joint categories. **(D)** Changes in the cumulative frequency of leading author keywords.

Classification by anatomical site showed that knee-related publications predominated, substantially exceeding publications concerning the elbow, shoulder, foot, hip, hand, or temporomandibular joint (Figure 5B). Further decomposition of anatomical expressions showed that the knee category was driven mainly by terms such as knee, knee joint, patellofemoral, and tibiofemoral, whereas the foot category primarily involved the ankle, metatarsophalangeal, and subtalar joints (Figure 5C). The distribution indicates that existing research is highly concentrated on the knee in terms of bibliographic visibility, but it does not represent the true prevalence or clinical importance of fibrosis in different joints.

### 3.5 Keyword co-occurrence, conceptual structure, and thematic evolution

The author-keyword co-occurrence network comprised four interconnected thematic clusters. Arthrofibrosis was the largest central node and was closely associated with total knee arthroplasty, stiffness, revision, and manipulation under anesthesia. The other clusters focused on the knee and arthroscopic treatment, anterior cruciate ligament injury and reconstruction, and inflammation, fibrosis, and shoulder-related disorders (Figure 6A). Factorial analysis revealed three main thematic clusters: stiffness after knee arthroplasty and its management, knee injury and ligament reconstruction, and inflammation- and fibrosis-related manifestations (Figure 6B).

**Figure 6:**
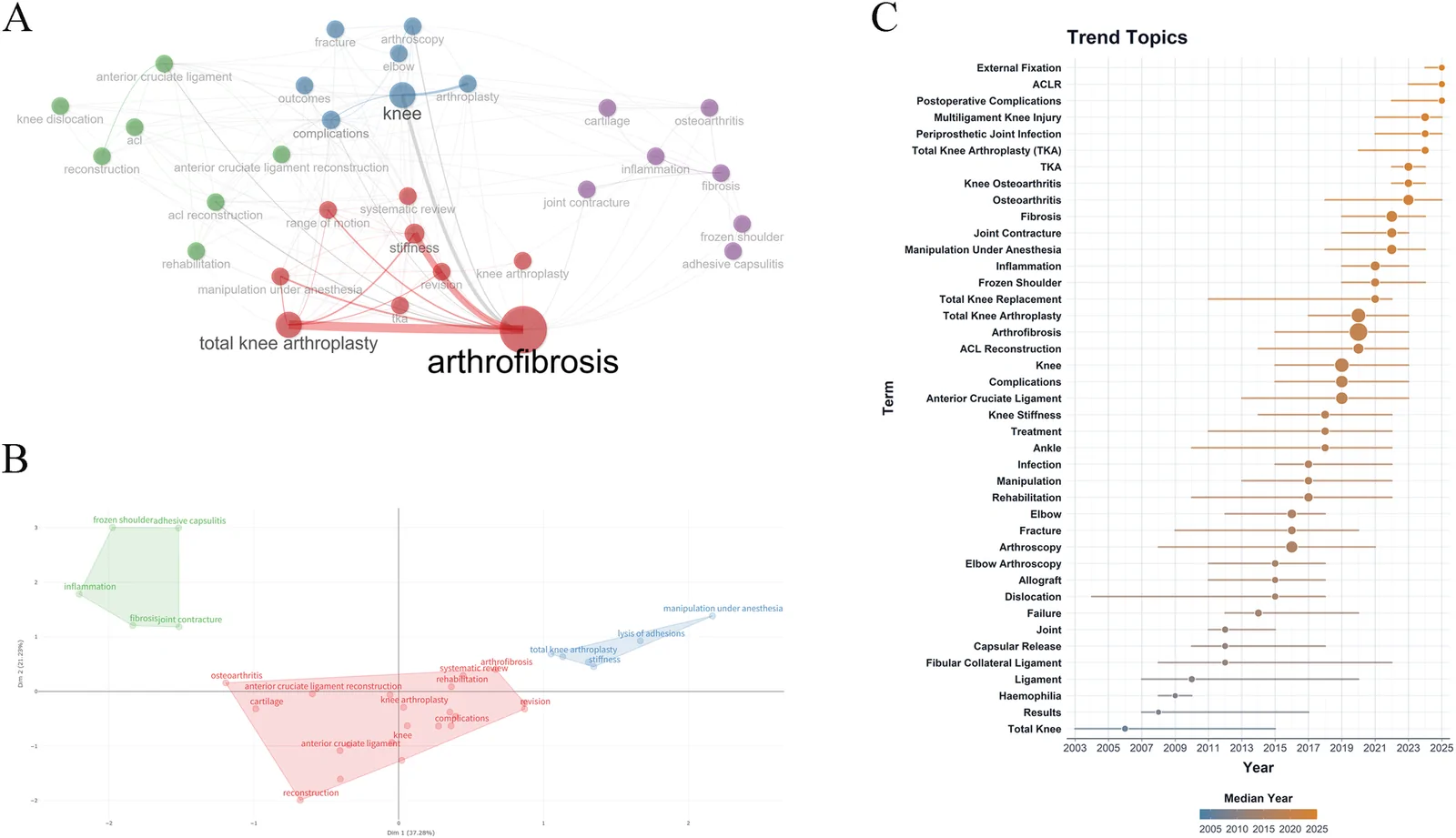
Keyword co-occurrence, factorial analysis, and trend topics based on author keywords. **(A)** Author-keyword co-occurrence network. **(B)** Factorial analysis of document–keyword relationships using multiple correspondence analysis, with spatial proximity indicating similar distribution patterns and color denoting thematic clusters. **(C)** Temporal distribution of trend topics.

Trend-topic analysis showed a shift from earlier broad themes involving joints, ligament injury, and arthroscopic treatment toward joint fibrosis, knee arthroplasty, and its complications. Periprosthetic joint infection, multiligament knee injury, postoperative complications, ACLR, and external fixation emerged as representative recent topics (Figure 6C). Overall, knee-related clinical problems formed the most prominent line of research in author keywords, while pathological processes such as fibrosis and inflammation formed a relatively distinct theme. Keyword visibility depends on authors’ choice of terms and cannot be used to determine the actual volume, quality, or level of evidence of mechanistic research.

### 3.6 Author, institutional, and country collaboration networks

The author collaboration network contained several relatively concentrated research teams. Collaboration groups centered on Matthew P. Abdel, Joaquín Sánchez-Sotelo, Mark E. Morrey, and André J. van Wijnen were comparatively large and densely connected (Figure 7A). The institutional collaboration network was centered on Mayo Clinic, Harvard-affiliated signing entities, and the University of Pennsylvania and contained several regional clusters (Figure 7B). In the country collaboration network, the United States was the most extensively connected hub, collaborating with Canada, China, Germany, the United Kingdom, Japan, Australia, and several European countries. China, Germany, the United Kingdom, and Canada were also important participants, although their network size and connectivity were generally lower than those of the United States (Figure 7C). The field exhibited a multilevel collaboration structure, but international links remained concentrated among a small number of core nodes.

**Figure 7:**
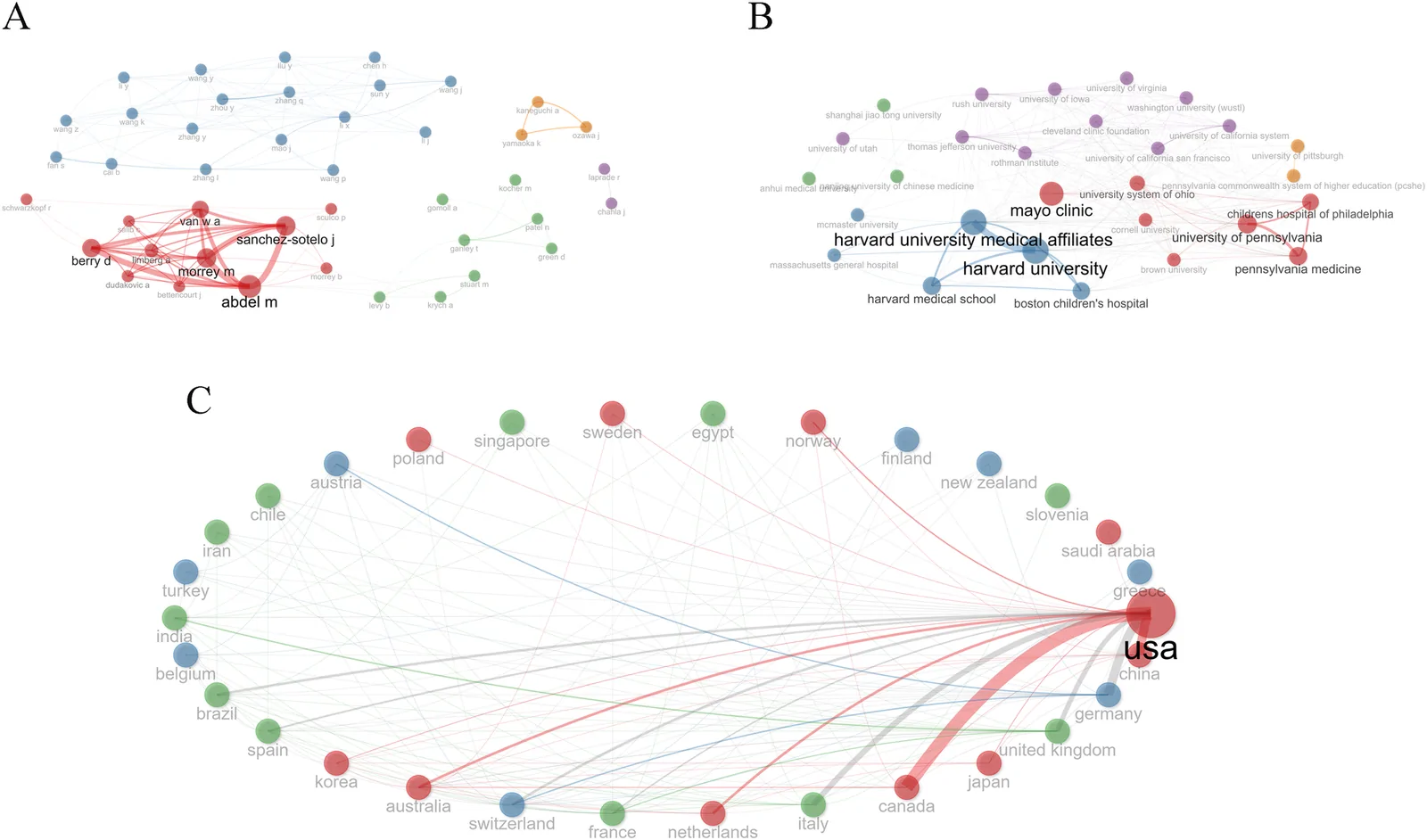
Author, institutional, and country collaboration networks. **(A)** Author collaboration network. **(B)** Institutional collaboration network. **(C)** Country collaboration network. Node size represents publication frequency, line thickness represents collaboration strength, and color represents cluster membership.

## 4 Discussion

Using a retrieval framework that combined fibrosis-specific terms with joint anatomy terms, this study constructed a corpus of 1,600 publications on joint fibrosis. Publication output increased overall, but the thematic and anatomical distributions did not expand evenly. The knee clearly predominated in the publication-level anatomical classification, while joint arthroplasty, anterior cruciate ligament reconstruction, management of postoperative stiffness, and functional recovery formed the principal knowledge trajectories. Research output and collaborative ties were also concentrated among a small number of countries, institutions, and author groups. Together, these findings indicate that the current knowledge base on joint fibrosis has been shaped primarily by knee-related clinical problems and core research networks.

The bibliographic predominance of the knee may first reflect its well-defined and frequently studied clinical contexts. Joint fibrosis after total knee arthroplasty can cause persistent motion restriction, functional decline, and repeat intervention, and relatively mature research pathways have developed around manipulation under anesthesia, arthroscopic lysis of adhesions, and revision surgery (28–31). Joint fibrosis after anterior cruciate ligament reconstruction similarly has a defined postoperative timeline and quantifiable outcomes; range of motion, reoperation, and patient-reported outcomes can be compared in cohort studies and systematic reviews (32). Thus, the continued accumulation of cases, outcome measures, and specialist publication platforms in knee arthroplasty and sports medicine may collectively increase the bibliographic visibility of knee joint fibrosis.

Mechanistic studies have identified TGF-β signaling, extracellular matrix remodeling, inflammatory mediators, and metabolic changes as processes associated with postoperative knee joint fibrosis (33, 34). However, the lower visibility of mechanism-related terms than of clinical topics among the author keywords in this study does not demonstrate that mechanistic research is insufficient in quantity or quality. Clinical studies tend to use procedure names, complications, and management approaches as keywords, whereas studies of molecular mechanisms do not necessarily include specific pathways among their author keywords. Keyword networks are therefore suitable for characterizing the explicit expression of research themes but cannot substitute for systematic evaluation of mechanistic evidence.

The elbow and shoulder had substantially fewer publications than the knee, but their research contexts differed. Post-traumatic elbow stiffness may result from capsular contracture, soft-tissue adhesions, heterotopic ossification, or osseous impingement, and recent reviews continue to emphasize the complexity of its etiology and treatment pathways (35–37). Shoulder research suggests that capsular fibroblast activation and inflammatory dysregulation may contribute to fibrosis, but frozen shoulder, postoperative shoulder stiffness, and pathological capsular fibrosis should remain conceptually distinct (38–40). These differences indicate that cross-joint comparisons should be grounded in clear diagnostic criteria and joint-specific contexts rather than grouping conditions solely on the basis of similar restricted-motion phenotypes.

Joint fibrosis, joint stiffness, and joint contracture frequently co-occur in the clinical literature but occupy different conceptual levels. Fibrosis describes a histological and pathobiological process, whereas stiffness and contracture describe states of motor function; the latter two may also result from mechanical, osseous, infectious, or neuromuscular causes. This boundary affects both search precision and the comparability of study populations and outcomes. Although the present study used fibrosis-specific concepts to define its corpus, it did not establish new diagnostic criteria. Future studies should report the diagnostic basis, anatomical location, precipitating factors, disease stage, and alternative causes excluded when specifying a disease entity involving fibrosis.

Different joints may share processes such as persistent inflammation, fibroblast activation, and extracellular matrix deposition, but they differ in capsular anatomy, functional demands, loading patterns, and common injury types. For example, joint fibrosis after hip arthroplasty and other capsular disorders of the hip have been clinically reported, but diagnostic and therapeutic evidence remains dispersed (41). The value of cross-joint research therefore lies primarily in comparing shared pathways with joint-specific differences and validating transferable diagnostic or research frameworks. Current bibliographic findings are insufficient to support treatment strategies applicable across different joints.

The United States occupied a central position in publication output, total citations, and the country collaboration network, while Mayo Clinic, Harvard-affiliated signing entities, and the University of Pennsylvania constituted major institutional nodes. This concentration may be related to stable sources of specialty cases, long-term follow-up systems, joint-surgery research platforms, and established interinstitutional collaboration. China showed rapid growth in Country Scientific Production, but corresponding-author country analysis indicated a relatively low proportion of internationally coauthored publications. Clustering within the author and institutional networks also suggests room to expand cross-team connections. Future studies should strengthen interinstitutional, international, and interdisciplinary collaboration and conduct multicenter cohorts and mechanistic-to-clinical translational research based on clearly defined diagnoses and outcome measures.

This study has several limitations. First, only Scopus and WoS were searched, and inclusion was restricted to English-language articles and reviews; publications from other databases, non-English literature, conference papers, guidelines, and other document types may therefore have been missed. The fibrosis-specific search improved thematic precision but may have omitted studies that discussed relevant pathological processes in the full text without explicitly using fibrosis-related terminology in the title, abstract, or keywords, and a small number of irrelevant records could not be completely excluded. Independent dual-reviewer screening of titles, abstracts, and full texts was not conducted for all 1,600 publications, nor was a formal search-sensitivity analysis performed. The findings should therefore be interpreted as the bibliographic structure within the prespecified search boundaries.

Second, anatomical classification relied on titles, abstracts, and author keywords and may have been affected by missing fields, ambiguous abbreviations, negation, multiple-joint assignment, and differences in classification granularity; no independently annotated gold standard has yet been established. Eighty-one publications could not be matched in OpenAlex, and author rankings and collaboration networks may contain a small degree of erroneous merging or splitting. Retaining institutions as separate signing entities may also have dispersed the output of related organizations. Citation-counting systems differ between WoS and Scopus, so the corresponding rankings are descriptive only. Annual publication counts were not normalized to total database output, and their growth cannot be directly interpreted as an increase in the relative scale of the field. Finally, bibliometric analysis cannot assess the quality of evidence, treatment effects, or risk of bias in individual studies and cannot replace a systematic review or meta-analysis addressing a specific clinical question.

## 5 Conclusion

Using a fibrosis-specific retrieval framework, this study characterized the bibliographic landscape of joint fibrosis research from 2000 to 2025. The available literature was highly concentrated on the knee and focused primarily on joint arthroplasty, ligament reconstruction, postoperative complications, and functional recovery. Future studies should further clarify the pathological and diagnostic boundaries of joint fibrosis, improve comparability across studies, and strengthen interinstitutional and international collaboration. These conclusions reflect bibliographic visibility within the prespecified search scope and cannot be directly interpreted as differences in prevalence, disease burden, or research quality across joints.

## Data Availability

The original contributions presented in the study are included in the article/Supplementary Material, further inquiries can be directed to the corresponding authors.
