# Supplementary material for: Joint fibrosis research from a multi-joint perspective: a bibliometric analysis, 2000–2025
Source: Front Surg. 2026 Sep 16;13:1885682. doi: 10.3389/fsurg.2026.1885682 (PMC13624128; doi:10.3389/fsurg.2026.1885682)
Supplement: Supplementary file 1 [file Table1.docx]

Table S1. MeSH Joints hierarchy and free-text anatomical terms used in the search

| **MeSH Joints hierarchy term** | **Free-text anatomical terms included in the search** |
| --- | --- |
| Acromioclavicular Joint | "acromioclavicular joint*" OR acromioclavicular |
| Atlanto-Axial Joint | "atlanto-axial joint*" OR "atlantoaxial joint*" OR atlantoaxial |
| Atlanto-Occipital Joint | "atlanto-occipital joint*" OR "atlantooccipital joint*" OR atlantooccipital |
| Bursa, Synovial | Not treated as an independent joint site |
| Cartilage, Articular | Not treated as an independent joint site |
| Elbow Joint | "elbow joint*" OR elbow |
| Foot Joints | "foot joint*" OR "toe joint*" OR ankle OR talocrural OR subtalar OR tibiotalar OR metatarsophalangeal OR interphalangeal |
| Hand Joints | "hand joint*" OR "finger joint*" OR wrist OR radiocarpal OR metacarpophalangeal OR interphalangeal |
| Hip Joint | "hip joint*" OR hip OR acetabulofemoral OR coxofemoral |
| Joint Capsule | Not treated as an independent joint site |
| Knee Joint | "knee joint*" OR knee OR tibiofemoral OR patellofemoral |
| Ligaments, Articular | Not treated as an independent joint site |
| Pubic Symphysis | "pubic symphysis" OR "symphysis pubis" |
| Sacroiliac Joint | "sacroiliac joint*" OR sacroiliac |
| Shoulder Joint | shoulder OR glenohumeral |
| Sternoclavicular Joint | "sternoclavicular joint*" OR sternoclavicular |
| Sternocostal Joints | "sternocostal joint*" OR sternocostal |
| Temporomandibular Joint | "temporomandibular joint*" OR temporomandibular OR TMJ |
| Zygapophyseal Joint | "zygapophyseal joint*" OR "facet joint*" |
